# Do Youth Dream of Gender Stereotypes? The Relationship among Gender Stereotyping, Support for Feminism, and Acceptance of Gender-Based Violence

**DOI:** 10.3390/ijerph20032439

**Published:** 2023-01-30

**Authors:** Carmen M. Leon, Eva Aizpurua

**Affiliations:** 1 School of Law, University of Castilla-La Mancha, 02071 Albacete, Spain; 2 NatCen Social Research, London EC1V 0AX, UK

**Keywords:** feminism, gender-based violence, gender stereotypes, young people, online survey

## Abstract

Although gender roles have continued to evolve, stereotypical perceptions about men and women persist. From a traditional perspective, men are viewed as aggressive, competitive, and dominant, whereas women are expected to be pretty, affectionate, and passive. The relevance of gender stereotypes lies in the way such expectations reinforce gender inequality and discrimination. Gender stereotyping is also linked to an increased acceptance of gender-based violence, as such conceptions are based on the premise that women are subordinate to men. The current study uses data from the Barometer on Youth and Gender, conducted by the *Centro Reina Sofía* in 2021 (*N* = 1201), to analyze the potential associations among gender stereotyping, support for feminism, and acceptance of gender-based violence among young people in Spain (15–29 years old). The results show that young people ascribe, to some extent, stereotypical characteristics to women and men and point to the existence of gender-based occupational stereotypes. Our results shed light on the role that gender stereotyping plays in support for feminism and the acceptance of gender-based violence. They also provide valuable information about the magnitude of gender-stereotypical perceptions among young men and women.

## 1. Introduction

There is broad consensus that stereotypes serve as an underlying justification for prejudice, which is an accompanying feeling—typically negative—towards individuals from specific social groups [1]. Stereotypes are general expectations that tend to represent members of particular social groups, influencing judgments regarding such members and the way individuals expect them to behave [2,3]. Gender stereotypes, in particular, are a structured set of shared beliefs within a culture or group of people concerning the attributes that are or ought to be possessed or the roles that are or should be performed by men and women [4]. Along with gender identity and ideology, gender stereotypes underlie discriminatory behaviors based on the categorization of a person as a man or a woman [5].

According to Social Role Theory, stereotypical beliefs about gender groups arise from the observation that each group performs different social roles, whereby the existence of different internal dispositions is inferred [6,7,8]. These beliefs, coupled with socialization and individual processes, favor the appearance of differentiated behaviors between men and women, which maintain gender stereotypes. With these beliefs, individuals construct gender roles that are responsive to cultural and environmental conditions yet appear, for individuals within a society, to be stable, inherent properties of men and women [8].

Gender stereotypes have detrimental consequences for men and women, as they limit the comprehensive development of individuals by influencing their preferences, skill development, aspirations, emotions, and performance [9]. Although the consequences of these stereotypes impact everyone, the evidence suggests that women experience greater negative effects, perpetuating gender inequality and discrimination, and increasing their risk of intimate-partner violence [10,11].

Given the role of gender stereotypes in discriminatory actions against women, this study examines the magnitude of gender stereotypes among young people in Spain. Accordingly, we analyze the attributes associated with women and men, and the extent to which gender-based occupational stereotypes exist among this population. In addition, we investigate the relationships between gender stereotyping, support for feminism, and the acceptance of gender-based violence.

### 1.1. Social Roles, Gender Stereotypes, and Objectification

According to Social Role Theory, gender stereotypes stem from the typical roles occupied by members of a group both in the home and at work [12]. In the domestic sphere, women have traditionally performed most routine domestic work and serve as primary caretakers. In the workplace, women have tended to be employed in people-oriented, service occupations rather than things-oriented, competitive jobs, which are traditionally occupied by men [13]. This contrasting distribution regarding social roles and the message it sends about what women and men are like have given rise to gender-stereotypical conceptions [12].

One of the most influential approaches to the conceptualization of gender stereotypes is the distinction between communal and agentic attributes. According to Bakan [14], communal characteristics, which are more strongly ascribed to women, describe a concern with the welfare of other people. In contrast, agentic characteristics, ascribed more strongly to men, describe a tendency for assertiveness, control, and confidence. In terms of social roles, women are more often valued for being affectionate, kind, sensitive, and empathetic, whereas men are expected to exhibit traits such as aggressiveness, self-confidence, dominance, and ambition [15,16].

The implicit impact of gender stereotypes has been documented in previous research showing that relational criteria dominate the ways in which people regard and evaluate women [17]. Individuals are inclined to evaluate women primarily based on their appearance rather than their accomplishments, which does not hold in the case of men [18]. As a result, physical appearance becomes a dominant factor in determining women’s worth, even in contexts where it should be irrelevant [19]. This association between women’s worth and their physical appearance leads to objectification [18,20]. In fact, it has been indicated that the perception of communality and agency might be a potential effect of the objectification process [21].

From the perspective of objectification, women are reduced to the status of “instruments” available for visual inspection, evaluation, and the pleasure of others [18]. Such objectification, along with gender stereotypes, has negative consequences for women [21]. For instance, it has been found that objectifying women degrades perceptions of their agency [22]. Research also shows that when women are objectified, they are seen as less than complete human beings [19]. Additionally, the “sexy woman” stereotype has been associated with a perceived lack of competence, and objectified women are viewed as less suited for high-status jobs [23]. However, such effects do not diminish the perceived qualities of men, even when they are evaluated based on their appearance [20].

### 1.2. Gender Stereotyping, Sexism, and Gender-Based Violence

Although Social Role Theory posits that changes in stereotypes would follow from the changes effected in the distribution of social roles [6], gender stereotyping still persists in Western countries. A study by Haines et al. [24] comparing data from the early 1980s and 2014, found that gender stereotyping remained strong and highly consistent over time. Despite differences in samples and time periods, the authors found little change in the extent to which beliefs about typical men and women were differentiated with respect to agentic and communal traits, male gender roles, male and female occupations, and male and female physical characteristics. However, results are mixed, and other studies have illustrated the dynamic nature of gender stereotypes and its evolution over time [25,26,27].

Since the propensity to evaluate people based on their sex is a pernicious social problem that might result in violations of human rights and fundamental freedoms [4], it is important to understand the factors associated with the persistence of gender stereotypes. Certain sociodemographic and sociocultural factors have been associated with gender stereotyping. For instance, it has been found that women, younger people, and those who have higher education levels have less stereotypical beliefs about how men and women should behave [28]. The evidence also reveals that individuals in less-populated areas tend to endorse more traditional gender-role beliefs [29]. More recently, Castillo-Mayén and Montes-Berges [9], in a study conducted among college students in Spain, found that gender, religiosity, age, marital status, and political orientation, in this order, had the greater effects on gender stereotyping. In addition, the authors found that apart from marital status, the effect of these variables was stronger on the attributes used to define women than those used to define men.

In contrast, feminism has been identified as one of the most important factors explaining counter-stereotypical behavior. In a study examining women’s attitudes towards gender stereotypes, van Breen et al. [30] found that a stronger identification with feminists was associated with the rejection of gender stereotypes among women. Along similar lines, when considering subliminal stereotypes, a general concern regarding sexism increases sensitivity to subliminal instances thereof [31]. These findings suggest that individuals who hold more egalitarian values may develop hyper-vigilance for gender-based threats and more sophisticated and diverse strategies for countering them [32].

Gender stereotypes play a role in perpetuating systemic gender inequalities by justifying existing norms [33]. While feminism is the advocacy of women’s rights on the basis of the equality of sexes, sexism is characterized by a belief in traditional gender role stereotypes and an inherent inequality between men and women. This prejudice against women is expressed through gender stereotypes, biased attitudes, and discrimination [34]. According to Swim et al. [35], the most traditional (“*old-fashioned*”) form of sexism is gender inequality occurring on the grounds of gender stereotyping. There is evidence linking adherence to traditional stereotypes to the justification of gender differences [36]. As a consequence, reducing gender stereotyping is an important step towards improving the ability to identify and confront sexism [37]. Although previous studies suggest the relevance of feminism and sexist beliefs when it comes to gender stereotyping, to our knowledge, no studies have analyzed the association between gender stereotyping and support for feminism (as a proxy for sexism [38,39]).

Sexism is also expressed in the form of gender-based violence against women. The European Institute for Gender Equality [40] defines this type of violence as violence inflicted on a woman because of her gender, or that affects women disproportionately. This definition frames such violence as a problem of men’s violence towards women, and roots its causes in the traditional gender inequality and power imbalances between men and women [41]. As can be noticed, the definition of gender-based violence is congruent with stereotypes about women and men, specifically, the notion that women are weak, in need of protection, and subordinate to men, who are powerful, strong, and self-reliant [42]. In fact, the evidence shows that gender stereotyping and sexist beliefs overlap with attitudes towards gender-based violence against women. For instance, ambivalent sexism has been found to be related with attitudes towards gender-based violence against women (for a review, see [43,44]). Specifically, those who score higher on sexism show a higher level of acceptance of gender-based violence [44]. Other attitudes and beliefs related to attitudes towards gender-based violence include traditional masculine roles [45] and egalitarian ideology [46].

### 1.3. Contextualizing the Study of Gender Stereotypes in Spain

In order to contextualize the study of gender stereotyping in Spain, it is important to consider the socio-political environment during the last decades. During Franco’s dictatorial regime in Spain (1939–1975), women’s rights were restricted. Many laws were rooted in the traditional division of labor between men and women, wherein the latter were expected to attend to the household and bear children. Franco imposed a traditional Catholic family model based on the subordination of women to their husbands. This resulted in the restriction of women to the domestic sphere, and their exclusion from a number of professions (e.g., magistrates, notaries, etc.) [47]. This hindered women’s access to education as well as vocational and professional life, and limited their rights in both public and private spheres. For instance, during this period, women needed permission to perform basic activities such as applying for a job or opening a bank account. However, the 1978 democratic constitution opened the door for Spanish regulation wherein women were guaranteed entirely equal rights [47].

Although men’s and women’s social conditions in Spain have been progressively converging, important disparities remain. The 2021 edition of the Gender Equality Index shows that the greatest gender inequality exists in the domain of time allocation, with Spain scoring one point lower (64) than the European Union (65) on a scale from 1 (full inequality) to 100 points (full equality) [48]. The domain of time measures gender inequalities in terms of the allocation of time spent on care-related and domestic work and social activities. The low score in Spain has been attributed to the persistent gender inequality with respect to the time spent on care and housework. Additionally, the Index reveals that since 2010, Spain’s scores have shown little progress in the domains of work (72 points in 2010 and 74 in 2021) and money (77 points in 2010 and 78 in 2021). Although the stability in the domain of money can also be found at the European level (79 points in 2010 and 82 in 2021), the figures in the domain of work evidence a decrease in the European Union’s scores (70 points in 2010 and 65 in 2021). The findings regarding Spain are consistent with some studies on gender stereotyping. For instance, a study conducted among a representative sample of the Spanish population whose objective was to analyze gender stereotypes using two different sets of data (1993 and 2001) found that the content of gender stereotypes had not changed substantially over the target period of time, even though the authors saw a noteworthy decrease in role-based stereotyping. Specifically, the results of this study confirmed the classical communal–agentic typology by finding a higher level of assignment of expressive-communal traits to women and of instrumental-agentic traits to men [49]. López-Sáez et al. found that the family facet attributed to women was the most acute and persistent role-stereotyping area. However, these findings are contrary to those found in a more recent study conducted in Spain, which showed that gender stereotypes have evolved from 1985 to 2018, parallel to the social changes in the situations of men and women [27].

## 2. Current Study

Since the realities of women and men have drastically changed in Spain in the last few decades, according to Social Role Theory, gender stereotypes should have changed as well. However, gender stereotypes might be resistant to change [24,28]. Given the importance of gender stereotypes with respect to discriminatory processes against women, it is essential to evaluate the magnitude of such beliefs among young people.

Based on Social Role Theory [6,7,8], we hypothesize that the prevalence of traditional stereotypical characteristics will be low (H_1_). At the same time, the existence of gender-based occupational stereotypes is expected, as they represent more subtle forms of stereotypical beliefs (H_2_). We anticipate that gender stereotyping and gender-based occupational stereotypes will be positively associated (H_3_). In addition, we hypothesize that individuals ascribing more traditional gender stereotypes to women and men will exhibit less support for feminism (H_4_) and higher acceptance of gender-based violence (H_5_).

## 3. Materials and Methods

### 3.1. Participants and Data Collection

The current study uses data from the Spanish Barometer on Youth and Gender, which was conducted by the *Centro Reina Sofía* in 2021. This study is conducted every two years and collects information on gender differences and inequalities, identities, affective experiences, and perceptions of gender inequality among young people aged 15–29 living in Spain. The 2021 edition constitutes the third wave, and focuses on gender stereotypes; relationships, inequalities, and discrimination; and harassment, gender-based violence, and intimate-partner violence.

The sample size was 1201 (50.6% women; *M* = 22.4 years old) and the participation rate was 36.6%. The scope of the survey was national, and the sample was extracted by the CINT panel via proportional allocation according to quotas for age and education level (ESOMAR information about the panel used is available here: https://es.cint.com/esomar28, accessed on 23 November 2022). Data were collected using a self-administered online questionnaire that was programmed in Spanish and took approximately 20–25 min to complete. Data collection took place during April and May of 2021. The microdata file and the questionnaire can be downloaded from the website of the *Centro Reina Sofía* (https://www.adolescenciayjuventud.org/bases-microdatos/, accessed on 23 November 2022). Further information about the study is available in Appendix A.

### 3.2. Measures

#### 3.2.1. Dependent Variables

*Support for feminism* (*α* = 0.84). Respondents were asked whether they agreed or disagreed with eight items, designed ad hoc, to measure support for feminism. The wording of all the items (translated into English) is presented in Table 5. The order of the items was randomized to minimize contextual effects and each item was measured on a 11-point agree/disagree scale. The response category “Don’t know/Prefer not to respond” was also offered. Four items were reverse-coded, and all the responses were averaged to create an index (range 0–10), in which higher scores indicated greater support for feminism.

*Acceptance of gender-based violence* (*α* = 0.82). Respondents were asked to report their levels of agreement with five items, designed ad hoc, measuring the acceptance of gender-based violence (“The following statements reflect some opinions about gender-based violence against women (physical, sexual, economic, control)”. On a scale of 0 “*I do not agree at all*” to 10 “*I fully agree*”, how would you rate your level of agreement with the following statements?”). The wording of the items is also available in Table 5. As with the support-for-feminism items, the order was randomized and the response category “Don’t know/Prefer not to respond” was offered. The item “gender-based violence is a very serious social problem” was reverse-coded, and the responses were averaged to create an index (range 0–10), in which higher scores indicated greater acceptance of gender-based violence.

#### 3.2.2. Independent Variables

*Gender stereotypes about women.* The endorsement of gender stereotypes about women was evaluated through the following question: “In your opinion, which of the following options best describe women? Read all the responses and choose up to three that you think describe them best.” The order of the response options was randomized to minimize response-order effects and included: (a) dynamic, active; (b) hard-working, studious; (c) responsible, prudent; (d) smart; (e) sensitive, affectionate; (f) calm; (g) dependent; (h) independent; (i) understanding; (j) worried about self-image, flirtatious; (k) possessive, jealous; (l) linked to home; (m) superficial; and (n) enterprising. The response category “Don’t know/Prefer not to respond” was also available and was exclusive.

*Gender stereotypes about men.* This question was asked after the question regarding female stereotypes and its content is as follows: “And which ones best describe men? Read all the responses and choose up to three that you think describe them best.” The response options were the same as those provided in the question about women and they were also randomized to minimize response-order effects.

To create an index for each variable where higher scores represent greater endorsement of stereotypes about women and men, we categorized the attributes based on which ones are considered stereotypical for women and men according to Bakan’s distinction [14] between communal and agentic attributes (see Table 1). Then, we created a count variable for each gender that ranged from 0 “*no stereotypical attribute selected*” to 3 “*all selected attributes were stereotypical*”.

*Gender-based occupational stereotypes* (*α* = 0.80). Respondents were asked to rate the suitability of various occupations for women and men (“Please, indicate how suitable are each of the following occupations for women and for men, with 0 being “*much better for women*” and 10 “*much better for men*”). The occupations were as follows: (a) welfare, health, and care of people; (b) education/teaching; (c) science and research; (d) computer science; (e) business management; and (f) engineering. For each item, the response category “Don’t know/Prefer not to respond” was available. The items “welfare, health, care of people” and “education/teaching” were reverse-coded so that higher scores represented greater stereotype endorsement. All responses were averaged to create an index (0–10), wherein higher scores indicate greater endorsement of gender-based occupational stereotypes. The exploratory factor analysis based on these items is available in Appendix B.

*Sociodemographic characteristics.* In the analyses, we also included some characteristics of the respondents, such as *gender*; *age* (in ranges); *nationality* (Spanish, non-Spanish); *political orientation* (measured using an 11-point left–right scale); *sexual orientation* (heterosexual or non-heterosexual); and *habitat size* (town or small city, mid-sized city, and large city with one million inhabitants or more).

### 3.3. Analytic Strategy

Statistical analyses were performed using Stata 16. We first examined the most frequently selected attributes to describe women and men as well as the suitability of occupations according to respondents’ gender. Differences between the groups were analyzed using chi-square and independent *t*-tests based on the characteristics of the variables. We also explored whether attributes were selected equally often to describe women and men (proportion tests). Then, we examined the distribution of the items composing the scales *support for feminism* and *acceptance of gender-based violence* and explored differences between men and women by using independent-samples *t*-tests. The relationships among key variables were also analyzed (Spearman’s Rho). Finally, we estimated multiple linear regression models to examine the associations among the variables under study. Variance Inflation Factors fell within appropriate limits, suggesting no multicollinearity problems (1.00 ≤ VIF ≤ 1.09).

Multiple imputation procedures were applied to handle missing data. The variables *political orientation* (13.1%), *support for feminism* (3.5%), and *acceptance of gender-based violence* (3.5%) showed the greatest proportion, while all other variables had less than 3.0% missing observations (for further information, see Appendix C). We used multiple imputation to create and analyze 30 multiply imputed datasets and incomplete variables were imputed under fully conditional specification. Outcome variables were included in the imputation model, but their values were not imputed, as the variables used in the imputation model are the same as the variables used in the analysis models.

## 4. Results

### 4.1. Attributes Used to Describe Women and Men

Table 2 displays the attributes selected to describe women and men, both for the full sample and disaggregated by respondents’ gender. For the full sample, the top three attributes used to describe women were *hard-working, studious* (41.5%), *smart* (38.6%), and *independent* (28.9%). When mapped with Table 1, it is noticeable that none of them are stereotypical characteristics for women. The attributes most selected to describe men were *independent* (33.7%), *hard-working, studious* (27.6%), and *enterprising* (25.4%), with two of them resembling traditional male stereotypes. As noted, two of the most selected attributes (i.e., *hard-working, studious*; and *independent*) overlapped for women and men.

When disaggregating the results by respondents’ gender, differences were found in nine of the fourteen attributes used to describe women. The greatest differences were found for *enterprising* (*V* = 0.20) and *independent* (*V* = 0.19), which were selected more often by women. Differences between the groups were also found in some of the traditional gender stereotypes attributed to women. For instance, it was found that men, compared to women, more frequently selected *sensitive, affectionate* (24.5% versus 18.5%) and *worried about self-image, flirtatious* (17.3% versus 10.4%) as the top attributes. Regarding the attributes selected to describe men, differences between genders were only found in three attributes: *dynamic, active*; *responsible, prudent*; and *superficial* (see Table 2). In this regard, women more often selected *dynamic, active* and *superficial* as top attributes to describe men, while the opposite was true for *responsible, prudent.*

To test whether the selection of attributes varies depending on whether they refer to women or men, tests assessing the equality of proportions were run. The results, presented in Table 3, reveal statistical differences for all attributes except for *calm*, which was selected similarly to describe women and men. In general, respondents more often selected traditional male stereotypes to describe men and traditional female stereotypes to describe women. This held true for all attributes identified as stereotypical for each gender except for *smart,* wherein more respondents selected this characteristic to describe women despite it being considered a male stereotype. The same is true for *dependent* and *superficial*, which were selected more often as top attributes to describe men even though they were identified as traditional female stereotypes.

The results from the count variables show that men selected more stereotypical attributes to describe women than women did (*t* = 4.56, *df* = 1192.86, and *p* ≤ 0.001), although the effect size was small (*d* = 0.26). Specifically, men selected 1.09 (*SD* = 0.88) stereotypical female attributes from the list, whereas women chose 0.86 (*SD* = 0.87). For the variable counting stereotypes about men, there were also significant differences based on gender (*t* = −2.21, *df* = 1191.65, and *p* = 0.027), but again, the effect size was small (*d* = −0.13). In this case, women selected more traditional male attributes than men (*M* = 1.13 and *SD* = 0.92; *M* = 1.02 and *SD* = 0.86, respectively). The correlation between both variables was positive and stronger for men (*rho* = 0.24, *p* ≤ 0.001; 95% CI 0.16–0.32) than women (*rho* = 0.12, *p* = 0.002; 95% CI 0.04–0.20), although the confidence intervals overlapped.

### 4.2. Gender Stereotyping around Occupations

Table 4 displays the descriptive statistics of the gender-based occupational stereotypes variables based on respondents’ gender. Across fields, less than 50% of young people indicated that the occupations are equally suited for men and women. This is especially true in *computer science* (40.3%) and *engineering* (42.7%), where respondents indicated that these occupations are generally more suited for men. No significant differences were found between women and men in terms of perceived suitability of occupations.

### 4.3. Gender Stereotyping, Support for Feminism, and Acceptance of Gender-Based Violence

Table 5 presents the descriptive statistics for the variables measuring *support for feminism* and *acceptance of gender-based violence*. Respondents leaned towards supporting feminism (*M* = 6.39; range 0–10), although women did so to a greater extent than men (*t* = −12.88; *df* = 1152.44; *p* ≤ 0.001; *d* = −0.76). On average, women scored around 1.5 points higher (*M* = 7.17; *SD* = 2.16) than men (*M* = 5.57; *SD* = 2.04) in the scale. Significant differences between women and men were found in all the items on the scale (see Table 5).

The results also show low acceptance of gender-based violence (*M* = 2.62; range 0–10), with men exhibiting a higher level of acceptance than women (*t* = 9.41; *df* = 1141.5; *p* ≤ 0.001; *d* = 0.56). On average, men scored over one point higher (*M* = 3.28; *SD* = 2.42) than women (*M* = 1.99; *SD* = 2.26) in the scale. Significant differences between the groups were found for all the items constituting the scale (see Table 5).

The correlations among the variables showed a moderate negative association between acceptance of gender-based violence and support for feminism (*rho* = −0.499; *p* ≤ 0.001). There was a weak positive correlation between gender stereotypes about men and gender stereotypes about women (*rho* = 0.167; *p* ≤ 0.001). The same is true for gender-based occupational stereotypes and gender stereotypes about men (*rho* = 0.106; *p* = 0.003) and women (*rho* = 0.111; *p* = 0.002). The results also revealed a weak negative correlation between gender stereotypes about men and acceptance of gender-based violence (*rho* = −0.195; *p* ≤ 0.001) as well as between gender stereotypes about women and support for feminism (*rho* = −0.142; *p* ≤ 0.001). A weak positive association between gender stereotypes about men and support for feminism was also found (*rho* = 0.114; *p* = 0.001). The gender-based occupational stereotypes scale was unrelated to both the acceptance of gender-based violence and support for feminism. No bivariate association was found between gender stereotypes about women and the acceptance of gender-based violence.

### 4.4. Correlates of Support for Feminism and Acceptance of Gender-Based Violence

To examine the correlates of support for feminism and the acceptance of gender-based violence, multiple linear regression models were estimated. The correlates on these models include personal characteristics (model 1) and variables related to gender stereotypes (model 2). Table 6 presents the results of these models. Six variables were significant in the models examining support for feminism: gender, sexual orientation, political orientation, and habitat size (personal variables), as well as gender stereotypes about men and women (count variables). These variables were significant in the models where they were included and most of them were associated with decreased support for feminism, except for gender and stereotypes about men. In this regard, women and individuals selecting more traditional male stereotypes to describe men were more supportive of feminism (*b* = 1.17, *p* ≤ 0.001 and *b* = 0.21, *p* = 0.002).

The models examining acceptance of gender-based violence are presented in Table 6. Gender, nationality, and political orientation (personal variables) as well as gender stereotypes about men were significant in the models where they were included. Most variables were associated with lower acceptance of gender-based violence, except for political orientation. In this regard, individuals leaning to the right were more accepting of gender-based violence.

The variables referring to gender stereotypes were, along general lines, significant in the models, but the percentage of explained variance barely increased after including them, thus suggesting limited explanatory power (see Table 6). We also examined the possibility that the relationship between gender-based occupational stereotypes and support for feminism/acceptance of gender-based violence was curvilinear (i.e., greater support for feminism and lower acceptance of gender-based violence among individuals with moderate scores in the scale regarding occupational gender stereotypes, which is suggestive of equal suitability). Accordingly, we included polynomial terms for the occupational stereotypes, but the resulting coefficients were not significant, providing robustness to the finding that occupational stereotypes play a small role in our models (see Appendix D).

## 5. Discussion

This study aimed to explore the magnitude of traditional gender stereotypes among young people in Spain. Accordingly, we examined stereotypical attributions for both women and men, as well as gender stereotypes for various occupations. This approach allowed us to uncover whether subtle form of gender stereotyping (i.e., the belief that women and men are not equally suitable for some occupations) are present among young people. At the same time, this study sheds light on the role of gender stereotyping in support for feminism and acceptance of gender-based violence. To our knowledge, this is one of the few studies analyzing the role of gender stereotyping in support for feminism—instead of sexist beliefs—and its influence on the acceptance of gender-based violence.

Our findings suggest that young people in Spain exhibit some level of stereotypical views of women and men. Although the distribution of the count variables indicate that youths ascribe low levels of traditional stereotypical characteristics, which supports our *first hypothesis,* it was also found that two of the most selected attributes to describe men (i.e., *independent* and *enterprising*) resemble traditional male stereotypes. Furthermore, the findings from the proportion tests reveal that the attributes were not equally chosen to describe men and women. Agentic attributes were more often chosen to describe men (e.g., *dynamic, active*; *independent*; and *enterprising*), while communal characteristics were more commonly selected to describe women (e.g., *responsible, prudent*; *sensitive, affectionate*; *understanding*; *worried about self-image, flirtatious*; and *linked to home*). The persistence of certain gender stereotypes was also found in a study conducted by Castillo-Mayén and Montes-Berges [9] among youths (18 to 29 years old) in Spain. The authors indicated that their results were expected, since a hierarchical system based on gender is part of Spanish society. They also alluded to the persistence of gender-based violence, the glass ceiling, and the perpetuation of gender roles and stereotypes in advertising to explain the maintenance of traditional gender stereotypes.

The results of the current study also show that men hold more stereotypical perceptions about women and that women hold more stereotypical perceptions about men. As noted by van Breen et al. [30], at the individual level, women might resist stereotypes by demonstrating that they—and by extension, their in-group—have counter-stereotypical attributes. This could explain why young women in this study more often selected traits not considered stereotypical for women (e.g., *smart; independent*). At the intergroup level, women who are motivated to challenge the devaluation of their own group might do so by either boosting evaluations of the in-group (in-group favoritism) or by rejecting the advantaged position of the out-group (out-group derogation). According to Eagly and Mladinic [50], individuals’ stereotypes about a social group reflect their attitude towards that group. Thus it seems plausible that gender-based subgroup perception conforms to the same biases seen in intergroup cognition in general, evidencing a strong intergroup bias [51].

Additionally, our results reveal that differences between women and men were more remarkable with respect to the attributes selected to describe women. Despite men still being associated with traditional male stereotypes (e.g., *independent* and *smart*) these same characteristics were also identified with women. This might be due to the diversification of social roles occupied by women since the 1970s, making it plausible that greater changes in female stereotypes have taken place [7,8]. The results showing that the top selected attributes used to describe women were *hard-working, studious,* and *smart* are consistent with a recent meta-analysis by Eagly et al. [52], highlighting that it is only in the competence sphere where gender equality has come to dominate individuals’ thinking about women and men. We was also found that men were more often assigned traditional female stereotypes such as *dependent* and *superficial* than women. These results are consistent with previous studies conducted in Spain, which have found that certain traditional stereotypes are attributed to the opposite gender [9,53].

An encouraging finding of the study is that some traditional female stereotypes, such as *superficial* (3.8%)*, linked to home* (4.5%)*, dependent* (6.1%), and *calm* (7.4%)*,* were not among the top attributes used to describe women. Furthermore, two of the most frequent attributes selected by the full sample to describe women and men coincided (i.e., *hard-working, studious,* and *independent*). Considering that gender stereotypes underline discriminatory processes against women, it is a positive finding that two of the most selected attributes to describe women and men overlapped.

Supporting our *second hypothesis,* the results of this study point to the existence of gender-based occupational stereotypes among young people in Spain. These beliefs are shared by both men and women and are particularly salient in the fields of *computer science* and *engineering*. As indicated in previous studies [54,55], while the percentage of women working in STEM (i.e., Science, Technology, Engineering, and Mathematics) fields are higher than decades ago, gender stereotypes that favor men as being more suitable for these fields persist, even among young populations. The absence of differences between genders suggests that both men and women are susceptible to internalizing these stereotypical beliefs.

Altogether, the findings of the current study reveal the existence of gender stereotypes in occupations, particularly in STEM fields. We know that gender stereotypes are impediments to women’s career advancement, promoting both gender bias in employment decisions and women’s self-limiting behavior [56]. Moreover, as the Social Role Theory posits, gender stereotypes will persist as long as gender segregation continues in society. Our results, however, provide limited support for this, given the weak association between gender stereotyping and gender-based occupation stereotypes (H_3_).

Regarding feminism, our findings show that young people in Spain have generally positive views of feminism. Consistent with previous research [57,58], women supported feminism to a greater extent than men. Although Spanish society is becoming more polarized with respect to gender issues, with antifeminism and traditional gender views being a common feature of the ideological underpinnings of populist radical right-wing parties [59], the items “feminism seeks to harm men” and “feminism has no real impact; it is only used as a political tool” were two of the ones that received the lowest levels of agreement.

Supporting our *fourth hypothesis,* the results of the current study point to the relevance of gender stereotyping in support for feminism. Those who held more gender stereotypes about women exhibited less support for feminism. This finding seems logical as stereotypes are used to legitimize intergroup inequality [60,61]; as such, the endorsement of gender stereotypes regarding women is likely to be negatively related with support for feminism. Our results are also aligned with those of van Breen et al. [30], who found that women who more strongly identified with feminists were more critical of gender stereotypes. At the same time, we found that youths who ascribed more traditional male stereotypes exhibited increased support for feminism.

Our results also indicate the relevance of sexual orientation, political orientation, and habitat size in support for feminism, with heterosexual individuals, youth leaning to the right, and those living in larger cities being less supportive of feminism. These results are, generally, consistent with previous research [57,58]. The finding regarding habitat size however runs contrary to the literature indicating that key predictors of pro-egalitarian and feminist attitudes include employment, younger age, higher education, and urbanicity [43].

The findings of the current study partially support our *fifth hypothesis*. The only variable related to gender stereotypes that was significant in the models examining acceptance of gender-based violence was gender stereotypes about men. Individuals who held more traditional male stereotypes were less likely to accept gender-based violence. At the same time, and consistent with previous research (for a review, see [44]), women and Spanish nationals were less accepting of gender-based violence. On the contrary, and also consistent with previous studies, individuals leaning to the right were more accepting of gender-based violence.

Despite low acceptance of gender-based violence, the items “although gender-based violence is wrong, it has always existed. It is unavoidable” and “gender-based violence is common within couples” congregated some support among young people (*M* = 3.30 and *M* = 2.58, respectively). This highlights the need to implement programs aimed at eradicating myths that are particularly rooted among youths. At the same time, men agreed to some extent with the statement “gender-based violence does not exist; it is an ideological invention” (*M* = 3.15, range 0–10), while the average dropped to *M* = 1.63 in the case of women.

Despite its contribution to the literature, this study has several limitations to consider. Firstly, the sample was non-probabilistic and might be subject to selection bias, thus limiting the generalizability of the findings. At the same time, weights were not available, limiting our ability to account and correct for non-response. The cross-sectional nature of the data and the fact that it only covers one country are also limitations of this study. Since we used secondary data, some variables identified as relevant in the literature (e.g., religiosity) could not be accounted for in the models. In addition, some of the attributes used to describe women and men were double-barreled (e.g., *worried about self-image, flirtatious*; *responsible, prudent*; *dynamic, active*), which could have increased measurement error. Another limitation was the imbalance in the traditional attributes used to describe men and women, as there were twice as many for women (see Table 1). Finally, given the complexity of the topic under study, mixed-methods research could further benefit the study of gender stereotypes and the processes linked to gender stereotyping.

## 6. Conclusions

Since gender stereotypes reinforce gender roles, social inequality, and gender discrimination [17], the design and implementation of initiatives to promote changes in traditional stereotypes are paramount, especially among younger populations. Further longitudinal and cross-cultural research is also needed to assess how contextual factors influence the endorsement of gender stereotypes over time and across different countries.

While our findings suggest that belief in some traditional female stereotypes (i.e., *linked to home, dependent,* and *calm*) is not pervasive among young people in Spain, gender stereotypes in occupational settings are prevalent. As Zitelny et al. [62] suggested, implicit stereotypes might play a more relevant role than explicit stereotypes in predicting behavior and career choices. This might explain why the current study found similarities between women and men in the competence sphere, but encountered gender stereotypes in occupational settings. Even though the belief that women and men are not as suitable to some occupations might be seen as a more subtle and implicit form of gender stereotyping, this preconceived idea still reflects stereotypical views of women and men [63]. In addition, gender stereotypes can limit women’s career advancement and promote self-limiting behaviors, thereby contributing to the glass ceiling phenomenon and the wage gap. For these reasons, implementing policies that promote gender equality, including mentoring programs and highlighting successful women in STEM, are potential steps towards making traditionally male-dominated occupations more accessible for women.

As gender stereotypes also impact how individuals search for romantic partners, the qualities they seek in them, and the way they engage in romantic relationships [17], it is important to understand the role of gender stereotyping in the acceptance of gender-based violence and support for feminism. The results from the current study point to the relevance of gender stereotyping with respect to support for feminism, although they explained a very small percentage of the variance. The results regarding acceptance of gender-based violence are even less consistent. Future research could provide greater insight into unexpected findings that might contribute to explain the relationship (or lack thereof) between traditional gender stereotypes about men and support for feminism and acceptance of gender-based violence.

## Figures and Tables

**Table 1:** Categorization of items based on stereotypical attributions for women and men.

Attribute	Women	Men	Neutral/Unclear
Dynamic, active		X	
Hard-working, studious			X
Responsible, prudent	X		
Smart		X	
Sensitive, affectionate	X		
Calm	X		
Dependent	X		
Independent		X	
Understanding	X		
Worried about self-image, flirtatious	X		
Possessive, jealous			X
Linked to home	X		
Superficial	X		
Enterprising		X	

Note: The categorization of the attributes is based on the distinction made by Bakan (1966) between communal and agentic attributes.

**Table 2:** Attributes selected to describe women and men by gender.

Variable	Full Sample (*N* = 1201)	Gender		*X^2^*	Cramer’s V
		Women (*n* = 605)	Men (*n* = 591)		
**Attributes Used to Describe Women**					
Dynamic, active	13.0% (155)	14.7% (89)	11.2% (66)	3.33	0.05
Hard-working, studious	41.5% (496)	48.6% (294)	34.2% (202)	25.60 ***	0.15
Responsible, prudent	26.6% (318)	28.4% (172)	24.7% (146)	2.13	0.04
Smart	38.6% (462)	41.8% (253)	35.4% (209)	5.25 *	0.07
Sensitive, affectionate	21.5% (257)	18.5% (112)	24.5% (145)	6.43 *	−0.07
Calm	7.4% (89)	5.8% (35)	9.1% (54)	4.88 *	−0.06
Dependent	6.1% (73)	5.0% (30)	7.3% (43)	2.80	−0.05
Independent	28.9% (346)	37.5% (227)	20.1% (119)	43.95 ***	0.19
Understanding	13.3% (159)	12.1% (73)	14.6% (86)	1.60	−0.04
Worried about self-image, flirtatious	13.8% (165)	10.4% (63)	17.3% (102)	11.78 ***	−0.10
Possessive, jealous	5.4% (65)	3.3% (20)	7.6% (45)	10.80 ***	−0.10
Linked to home	4.5% (54)	4.1% (25)	4.9% (29)	0.42	−0.02
Superficial	3.8% (45)	1.3% (8)	6.3% (37)	20.13 ***	−0.13
Enterprising	19.7% (236)	27.4% (166)	11.8% (70)	45.90 ***	0.20
**Attributes to Describe Men**					
Dynamic, active	23.8% (284)	28.3% (171)	19.1% (113)	13.81 ***	0.11
Hard-working, studious	27.6% (330)	27.9% (169)	27.2% (161)	0.07	0.01
Responsible, prudent	20.2% (241)	17.7% (107)	22.7% (134)	4.62 *	−0.06
Smart	24.6% (294)	22.6% (137)	26.6% (157)	2.48	−0.05
Sensitive, affectionate	5.0% (60)	4.8% (29)	5.3% (31)	0.13	−0.01
Calm	8.6% (103)	9.4% (57)	7.8% (46)	1.02	0.03
Dependent	15.9% (190)	16.5% (100)	15.2% (90)	0.38	0.02
Independent	33.7% (403)	34.4% (208)	33.0% (195)	0.26	0.02
Understanding	7.4% (88)	6.1% (37)	8.6% (51)	2.77	−0.05
Worried about self-image, flirtatious	7.9% (95)	9.3% (56)	6.6% (39)	2.89	0.05
Possessive, jealous	12.1% (145)	13.1% (79)	11.2% (66)	1.00	0.03
Linked to home	2.5% (30)	1.8% (11)	3.2% (19)	2.38	−0.05
Superficial	23.2% (278)	25.8% (156)	20.6% (122)	4.43 *	0.06
Enterprising	25.4% (304)	27.8% (168)	23.0% (136)	3.57	0.06

Note: The traditional stereotypes used to describe women are colored in pink, whereas the traditional ones for men are in blue. The sum of men and women do not add up to 1201 (full sample) because there were five cases in which participants left the gender question blank. Percentages do not add up to 100% because respondents could select up to three attributes. * *p* ≤ 0.05 and *** *p* ≤ 0.001.

**Table 3:** Attributes selected to describe women and men: two-sample tests of proportions.

Variable	For Women *M* (*SE*)	For Men *M* (*SE*)	*z*	*p*
Dynamic, active	0.13 (0.01)	0.24 (0.01)	−6.81	≤0.001
Hard-working, studious	0.41 (0.01)	0.27 (0.01)	7.13	≤0.001
Responsible, prudent	0.27 (0.01)	0.20 (0.01)	3.66	≤0.001
Smart	0.38 (0.01)	0.24 (0.01)	7.38	≤0.001
Sensitive, affectionate	0.22 (0.01)	0.05 (0.01)	11.96	≤0.001
Calm	0.07 (0.01)	0.09 (0.01)	−1.05	0.295
Dependent	0.06 (0.01)	0.16 (0.01)	−7.65	≤0.001
Independent	0.29 (0.01)	0.34 (0.01)	−2.51	0.012
Understanding	0.13 (0.01)	0.07 (0.01)	4.69	≤0.001
Worried about self-image, flirtatious	0.14 (0.01)	0.08 (0.01)	4.58	≤0.001
Possessive, jealous	0.05 (0.01)	0.12 (0.01)	−5.84	≤0.001
Linked to home	0.04 (0.01)	0.02 (0.01)	2.67	≤0.001
Superficial	0.04 (0.01)	0.23 (0.01)	−13.86	≤0.001
Enterprising	0.20 (0.01)	0.25 (0.01)	−3.32	≤0.001

Note: The traditional stereotypes used to describe women are colored in pink, whereas the traditional ones for men are in blue. *M* = mean; *SE* = standard error.

**Table 4:** Gender-based occupational stereotypes by gender.

Variable	Full Sample % Selecting the Middle Point (*n*)	Gender		*t*
		Women % Selecting the Middle Point (*n*)	Men % Selecting the Middle Point (*n*)	
Welfare, health, care of people	45.5% (529) *M* = 4.26, *SD* = 2.37 (range 0–10)	52.7% (308) *M* = 4.36, *SD* = 2.28 (range 0–10)	38.4% (220) *M* = 4.16, *SD* = 2.47 (range 0–10)	−1.47
Education/teaching	47.9% (557) *M* = 4.49, *SD* = 2.17 (range 0–10)	52.5% (308) *M* = 4.54, *SD* = 2.07 (range 0–10)	42.9% (246) *M* = 4.45, *SD* = 2.28 (range 0–10)	−0.74
Science and research	47.6% (552) *M* = 5.26, *SD* = 2.22 (range 0–10)	53.9% (315) *M* = 5.32, *SD* = 2.14 (range 0–10)	41.1% (235) *M* = 5.21, *SD* = 2.32 (range 0–10)	−0.82
Computer science	40.3% (469) *M* = 6.02, *SD* = 2.24 (range 0–10)	47.0% (276) *M* = 6.07, *SD* = 2.12 (range 0–10)	33.5% (192) *M* = 5.97, *SD* = 2.35 (range 0–10)	−0.80
Business management	48.7% (562) *M* = 5.44, *SD* = 2.20 (range 0–10)	54.3% (318) *M* = 5.37, *SD* = 2.08 (range 0–10)	42.9% (243) *M* = 5.53, *SD* = 2.32 (range 0–10)	1.28
Engineering	42.7% (493) *M* = 5.84, *SD* = 2.27 (range 0–10)	49.7% (291) *M* = 5.86, *SD* = 2.11 (range 0–10)	35.2% (200) *M* = 5.82, *SD* = 2.44 (range 0–10)	−0.28

Note: Only the percentage of respondents who selected the middle response option (5 in a range from 0 “*much better for women*” to 10 “*much better for men*”) are displayed in the table. Full data is available upon request from the authors. *M* = median, *SD* = standard deviation.

**Table 5:** Descriptive statistics of the support for feminism and acceptance of gender-based violence variables by gender.

Variable	Full Sample *M* (*SD*)	Gender		*t*	Cohen’s d
		Women *M* (*SD*)	Men *M* (*SD*)		
**Support for Feminism** (range 0–10)					
Support for Feminism (α = 0.84)	6.39 (2.25)	7.17 (2.16)	5.57 (2.04)	−12.88 ***	−0.76
Individual items (range 0–10):					
Feminism is necessary to achieve real equality between men and women	5.90 (3.43)	6.84 (3.18)	4.91 (3.40)	−9.93 ***	−0.59
Feminism seeks to harm men (R)	3.28 (3.34)	2.43 (3.22)	4.17 (3.23)	9.13 ***	0.54
Feminism seeks to overcome traditional barriers to allow women to access equality	6.58 (3.14)	7.49 (2.83)	5.64 (3.17)	−10.37 ***	−0.62
Feminism has no real impact; it is only used as a political tool (R)	3.90 (3.22)	3.37 (3.17)	4.45 (3.19)	5.71 ***	0.34
Feminism is essential to achieve a just society	6.20 (3.27)	7.13 (3.05)	5.24 (3.21)	−10.12 ***	−0.60
Feminism does not care about the real problems of women (R)	3.88 (3.31)	3.17 (3.24)	4.62 (3.22)	7.55 ***	0.45
Feminism must involve both women and men	6.77 (3.07)	7.21 (3.07)	6.32 (3.19)	−4.81 ***	−0.29
Feminism is not necessary because equality between men and women already exists (R)	3.31 (3.37)	2.32 (3.37)	4.36 (3.33)	10.66 ***	0.63
**Acceptance of Gender-based Violence** (range 0–10)					
Acceptance of Gender-based Violence (α = 0.82)	2.62 (2.43)	1.99 (2.26)	3.28 (2.42)	9.41 ***	0.56
Individual items (range 0–10):					
Gender-based violence is common within couples	2.58 (3.13)	2.20 (3.04)	2.97 (3.17)	4.18 ***	−0.25
If gender-based violence is of low intensity, it is not a problem for the couple’s relationship	2.26 (3.02)	1.53 (2.64)	3.02 (3.18)	8.55 ***	−0.51
Although gender-based violence is wrong, it has always existed. It is unavoidable	3.30 (3.35)	2.75 (3.24)	3.87 (3.36)	5.73 ***	−0.34
Gender-based violence is a very serious social problem (R)	7.45 (3.15)	8.22 (2.86)	6.64 (3.24)	−8.73 ***	−0.52
Gender-based violence does not exist; it is an ideological invention	2.37 (3.21)	1.63 (2.83)	3.15 (3.40)	8.13 ***	0.49

Note: *M* = median, *SD =* standard deviation. (R) = reversed item. *** *p* ≤ 0.001.

**Table 6:** Correlates of support for feminism and acceptance of gender-based violence (multiple linear regression models).

Variable	Support for Feminism				Acceptance of Gender-Based Violence			
	Model 1		Model 2		Model 1		Model 2	
	*b*	[95% CI]	*b*	[95% CI]	*b*	[95% CI]	*b*	[95% CI]
Gender (women)	1.22 ***	[0.99, 1.46]	1.17 ***	[0.94, 1.40]	−0.99 ***	[−1.25, −0.72]	−0.94 ***	[−1.20, −0.68]
Age (ref. 15–19 years old)								
20–24 years old	0.28	[−0.00, 0.57]	0.25	[−0.03, 0.53]	−0.31	[−0.63, 0.02]	−0.25	[−0.57, 0.07]
25–29 years old	−0.09	[−0.37, 0.19]	−0.09	[−0.36, 0.19]	−0.24	[−0.56, 0.07]	−0.19	[−0.51, 0.12]
Nationality (Spanish)	0.18	[−0.15, 0.51]	0.18	[−0.15, 0.51]	−0.48 *	[−0.85, −0.10]	−0.49 **	[−0.86, −0.12]
Sexual orientation (heterosexual)	−0.42 **	[−0.71, −0.13]	−0.44 **	[−0.73, −0.14]	−0.15	[−0.48, 0.18]	−0.05	[−0.39, 0.28]
Political orientation (left–right)	−0.34 ***	[−0.39, −0.29]	−0.33 ***	[−0.38, −0.28]	0.34 ***	[0.28, 0.40]	0.33 ***	[0.27, 0.39]
Habitat (ref. town or small city)								
Mid-sized city with 10,000 inhabitants or less	−0.33 *	[−0.61, −0.05]	−0.30 *	[−0.58, −0.03]	0.32	[−0.00, 0.64]	0.28	[0.04, 0.60]
Large city with 1M inhabitants or more	−0.38 **	[−0.66, −0.10]	−0.38 **	[−0.66, −0.11]	0.09	[−0.24, 0.41]	0.11	[−0.21, 0.43]
Gender stereotypes about men			0.21 **	[0.08, 0.34]			−0.46 ***	[−0.61, −0.32]
Gender stereotypes about women			−0.18 **	[−0.31, −0.05]			0.03	[−0.12, 0.17]
Gender-based occupational stereotypes			0.02	[−0.08, 0.12]			0.03	[−0.08, 0.14]
*F*	53.05 ***		40.56 ***		30.67 ***		26.63 ***	
*N*	1159		1159		1159		1159	
*Adjusted R-square*	0.27		0.28		0.18		0.20	

Note: *b* = unstandardized coefficient; CI = confidence intervals; * *p* ≤ 0.05, ** *p* ≤ 0.01, and *** *p* ≤ 0.001.

## Data Availability

The microdata file is available to download from the website of the *Centro Reina Sofía*.

## Appendix A

**Table A1:** Information about the 2021 Barometer on Youth and Gender.

Spanish Barometer on Youth and Gender	
Institution in charge	Centro Reina Sofía (https://www.adolescenciayjuventud.org/que-es-el-crs/ accessed on 1 November 2022)
Survey frequency	Once every two years
Population	Young people between 15 and 29 years old
Geographical coverage	Spain
Panel provider	CINT (https://es.cint.com/ accessed on 1 November 2022)
Sampling approach	Non-probabilistic Proportional allocation—quotas for age and education
Achieved sample size	1201
Participation rate	36.6%
Fieldwork period	April and May 2021
Main topics covered	Gender stereotypes; relationships, inequalities, and discrimination; harassment, gender-based violence, and intimate partner violence
Survey mode	Online
Survey length	20–25 min
Data access	https://www.adolescenciayjuventud.org/bases-microdatos/ accessed on 1 November 2022

## Appendix B

The factorial structure of the gender-based occupational stereotype items was examined by running an exploratory factor analysis (EFA). The principal factor method was the estimation technique used to analyze the correlation matrix. The number of factors was evaluated using the criterion of eigenvalues greater than one. The results suggest a one-factor structure accounting for 94.2% of variance. As displayed in Table A2, all items loaded in the same factor, with factor loadings between 0.48 and 0.73.

**Table A2:** Factor loadings of the six items measuring gender-based occupational stereotypes.

Item	Factor 1	Uniqueness
Welfare, health, care of people	0.54	0.71
Education/teaching	0.48	0.77
Science and research	0.71	0.49
Computer science	0.67	0.55
Business management	0.68	0.53
Engineering	0.73	0.47

## Appendix C

**Table A3:** Missing data.

Variable	% (*n*)
Political orientation	13.1% (157)
Support for feminism	3.5% (42)
Acceptance of gender-based violence	3.5% (42)
Gender-based occupational stereotypes	2.8% (34)
Sexual orientation	2.6% (31)
Habitat	2.0% (24)
Gender	0.4% (5)
Nationality	0.2% (2)

Note: The variables “age”, “gender stereotypes about women”, and “gender stereotypes about men” had no missing data and are excluded from this table.

## Appendix D

**Table A4:** Correlates of support for feminism and acceptance of gender-based violence (multiple linear regression models with a polynomial term for gender-based occupational stereotypes).

Variable	Support for Feminism		Acceptance of Gender-Based Violence	
	*b*	[95% CI]	*b*	[95% CI]
Gender (women)	1.17 ***	[0.93, 1.40]	−0.94 ***	[−1.20, −0.68]
Age (ref. 15–19 years old)				
20–24 years old	0.25	[−0.03, 0.53]	−0.25	[−0.57, 0.07]
25–29 years old	−0.09	[−0.36, 0.19]	−0.19	[−0.51, 0.12]
Nationality (Spanish)	0.18	[−0.15, 0.51]	−0.49 **	[−0.86, −0.12]
Sexual orientation (heterosexual)	−0.45 **	[−0.74, −0.16]	−0.05	[−0.38, 0.28]
Political orientation (left–right)	−0.33 ***	[−0.38, −0.28]	0.33 ***	[0.27, 0.39]
Habitat (ref. town or small city)				
Mid-sized city with 10,000 inhabitants or less	−0.30 *	[−0.58, −0.02]	0.28	[−0.04, 0.60]
Large city with 1M inhabitants or more	−0.38 **	[−0.66, −0.10]	0.11	[−0.21, 0.43]
Gender stereotypes about men	0.20 **	[0.07, 0.33]	−0.46 ***	[−0.61, −0.31]
Gender stereotypes about women	−0.18 **	[−0.32, −0.05]	0.03	[−0.12, 0.17]
Gender-based occupational stereotypes	0.62 *	[0.01, 1.24]	−0.06	[−0.77, 0.65]
Gender-based occupational stereotypes^2	−0.05	[−0.10, 0.00]	0.01	[−0.05, 0.07]
*F*	37.63 ***		24.37 ***	
*N*	1159		1159	
*Adjusted R-square*	0.28		0.20	

Note: *b* = unstandardized coefficient, CI = confidence intervals * *p* ≤ 0.05, ** *p* ≤ 0.01, and *** *p* ≤ 0.001.
